# Adoption and Initial Implementation of a National Integrated Care Programme for Diabetes: A Realist Evaluation

**DOI:** 10.5334/ijic.5815

**Published:** 2022-07-14

**Authors:** Kate O’Neill, Fiona Riordan, Emmy Racine, Marsha Tracey, Chrysanthi Papoutsi, Patricia M. Kearney, Sheena M. McHugh

**Affiliations:** 1 School of Public Health, University College Cork, Cork, IE; 2 Eli Lilly and Company, Cork, IE; 3 Nuffield Department of Primary Care Health Sciences, University of Oxford, GB

**Keywords:** diabetes, integrated care, realist evaluation

## Abstract

**Background:**

The implementation of models of integrated care for chronic conditions is not well understood. We conducted a realist evaluation to determine how and why the implementation of the National Diabetes Programme in Ireland worked (or not).

**Methods:**

Documentary analysis and qualitative interviews with a purposive sample of national stakeholders (n = 19), were used to develop an initial theory on expected programme delivery. We refined this theory using semi-structured interviews (n = 38) with professionals from different clinical disciplines involved in programme implementation.

**Results:**

Locally important contexts facilitating implementation included staff experience of delivering diabetes care, capacity, and familiarity with the intended purpose of new clinical posts. The extent to which integrated care was adopted and implemented depended on judgements made by professionals working in these contexts; specifically, judging the relative advantage of the programme and whether to engage in negotiations to legitimize their new roles in diabetes care.

**Conclusions:**

Our results highlight the need for adequate preparatory work to raise awareness of and support new roles to implement integrated care, clarification on the core components of new care models, and the development of local service infrastructures to support integrated care.

## Background

Worldwide, the increasing burden of chronic disease is leading to health system reforms, including strengthening primary care and increasing the provision of specialist support in the community [1]. Integrated care has become a pillar of chronic disease management reform internationally as a way to address service fragmentation, improve patient experience, and achieve better efficiency and value from healthcare systems [2,3,4,5]. Diabetes is often used as an exemplar chronic condition to study health service reforms such as integrated care as effective diabetes management requires the involvement of healthcare practitioners (HCPs) from different disciplines and settings [6,7].

Over the past four decades the prevalence of diabetes has increased substantially; in 2014 it was estimated that 8.5% adults globally have the condition [8]. Should trends in diabetes prevalence and mortality continue, it is estimated that by 2030, the global economic burden of diabetes will increase to $2.5 trillion or 2.2% of global GDP [7]. Diabetes-specific integrated care programmes have been implemented across Europe [9,10,11,12,13,14,15,16,17,18] as well as in high income countries such as Australia [19,20,21] and Singapore [22]. These programmes often comprise protocols to guide patient stratification according to risk/need [10,11,13,14,15], and the introduction of new staff to support multidisciplinary working [10,12,13,16,17,18]. In Ireland, a National Diabetes Programme (NDP) was established in 2010; one of several clinical care programmes set up as part of a larger reform process [23]. The overarching goal of the NDP was to standardise diabetes care delivery. Among other reforms, the NDP introduced national models of integrated of care for routine diabetes management across primary, secondary, and tertiary settings and for the management of diabetic foot disease. Historically, diabetes care in Ireland has been hospital-centric with a disconnect between secondary and primary care services in how they are funded, managed, and resourced [24]. There was a deficient in access to allied health services such as podiatry, and diabetes nurse specialists [DNS] were primarily hospital-based [25]. Some alternatives to hospital-based management have developed in the past few years; shared care between general practitioners [GPs] and hospitals, and structured primary care-led management [26,41].

The process by which models of integrated care are implemented is not well understood [27,28,29,30,31]; specifically, why these models work in some circumstances and not others [10,32,33]. There is growing emphasis on theory-based evaluations which are better equipped to deal with the complexity of introducing such multi-component interventions, like integrated care for chronic conditions such as diabetes, into complex and dynamic health systems [34]. Theory-based evaluations address some of the limitations of more traditional or quasi-experimental research designs which do not adequately factor in the role of context in the delivery of health system interventions [35,36]. The realist evaluation approach, developed by Pawson and Tilley, is a type of theory-based evaluation interested in explaining what works, for whom, how and why, in different contexts. The approach tries to determine the mechanisms and the contextual factors that bring about change, rather than focusing on specific endpoints [37,38,39,40].

We carried out a realist evaluation during the early stages of the NDP implementation to examine *how and why* it worked (or not) in different settings and for different groups across the country, specifically;

1. How was the programme implemented and by what mechanisms did its components lead to different outcomes?
2. What are the important contexts that determined whether these mechanisms produced intended (or unintended) outcomes?

## Methods

### National Programme for Diabetes

**Figure 1:**
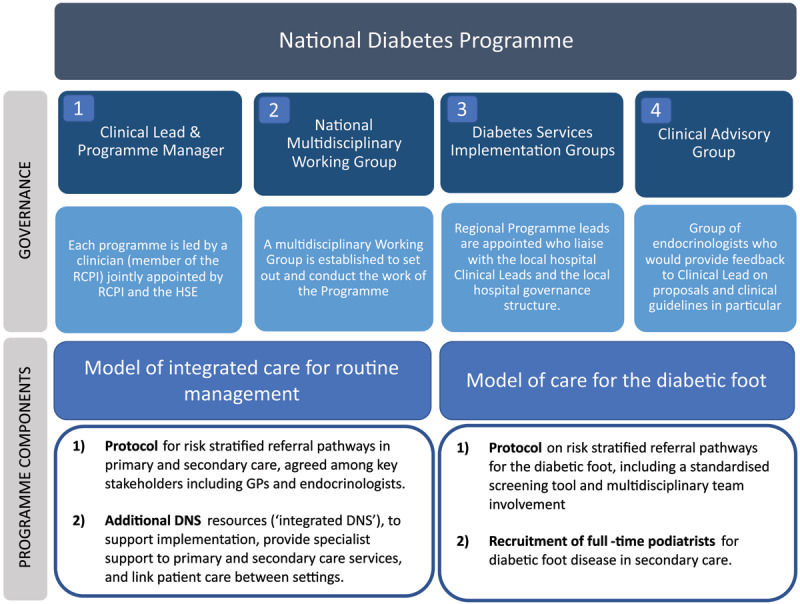
National Diabetes Programme.

The overarching goal of the NDP is to standardise diabetes care delivery. It was led by a national clinical lead and programme manager supported by representatives from various clinical disciplines: 1) a multidisciplinary working group, 2) advisory group, and 3) regional implementation groups (Figure 1). Once the programme was implemented into the health system, there were liaison structures in place between local hospitals and GPs represented by the implementation groups.

As part of national reforms, governance structures and programme components were developed by the Health Service Executive and the Royal College of Physicians in Ireland for each of the new clinical programmes (Figure 1). In line with their goal to develop standardised patient pathways and evidence-based models of care, the NDP working group developed national models of integrated care for the routine management of diabetes and diabetic foot disease [43], and the addition of new staff resources (Figure 1, Additional File 1).

### Design

**Figure 2:**
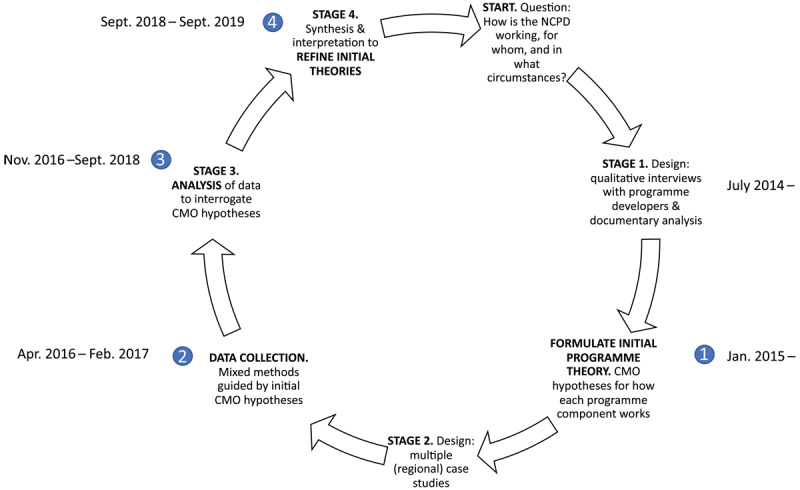
Realist research cycle adapted with permission from Marchal et al. [34].

This evaluation follows the research stages originally outlined by Pawson and Tilley [40] (Figure 2). Detailed methods can be found in the research protocol [44]. The evaluation was conducted in line with quality standards and reported based on realist evaluation publication standards. Additional File 2 outlines where the methods map to the reporting standards for realist evaluations (RAMESES II) [45]. Details on sampling, recruitment and data collection are outlined in the protocol [44].

### Formulating initial programme theories

#### Stage 1: Data collection and analysis to formulate initial programme theories

Stage one involved: 1) documentary analysis of policy documents, national working group presentations, and national service plans (2011–2014) to establish the official programme theory and, 2) concurrent qualitative interviews (conducted by MT) with a purposive sample of stakeholders from the NDP working group involved at a national level in the design, development, and management of the programme. Some participants were implementers within their own local diabetes service in addition to being involved in programme design at national level. Analysis focused on building plausible Context Mechanism Outcome Configuration (CMOC) statements that explained how the programme was expected to work. In addition to discussing planned implementation and expected outcomes (official programme theory), participants discussed their own experience of implementation, and locally perceived outcomes in their area, and the barriers encountered. This information was also used later in Stage 4 to refine the initial programme theory.

### Refining initial programme theories

#### Stage 2: Data collection to test initial programme theories

A case study design was used, informed by the initial programme theory. Stage two involved semi-structured interviews with professionals in each case, defined as a geographical area within one of the four administrative regions in the national health service. Cases were sampled based on two criteria: 1) receipt of a NDP component (i.e., integrated DNS and/or podiatrist), and 2) the presence or absence of an existing diabetes initiative [44]. Within each case, professionals were purposively sampled to represent different disciplines working in that geographical area: endocrinologist, GP, practice nurse (PN), DNS/integrated DNS, podiatrists, local implementation group member.

A topic guide was developed based on the initial programme theories developed during stage one. The topic guide was tailored to the stakeholder group being interviewed. Drawing on the principles of theory-driven interviewing [42], participants were invited to explain how their experience fit (or did not fit) with our initial theory and reflect on what may explain the outcomes in their area. During the interview, the interviewer (MT, FR, KON) had an active and explicit role in explaining the contexts and outcomes of interest, to ensure a shared understanding of the terminology and purpose of the question (i.e., to elucidate how the programme was working). The topic guide was piloted with a convenience sample of one GP and two PNs familiar with or in receipt of most programme components. With the written consent of participants, interviews were digitally recorded, transcribed verbatim and imported into NVivo 11 software.

#### Stage 3: Analyse data to interrogate theories and assess outcomes

During analysis, we adopted a retroductive approach, moving between inductive and deductive analysis, as well as following insights and hunches [46]. Using programme outcomes as the starting point, transcripts were analysed to 1) link an outcome to the response (mechanism) which most likely contributed to it, and then 2) linking this mechanism to the circumstances that triggered it (context). Mechanisms were often implicit therefore inference was required to determine whether the data were functioning as a C, M or O and the relationships between them. To identify potential patterns, Context-Mechanism-Outcome Configurations (CMOCs) from each transcript, along with supportive quotes, were tabulated in a Word document. For each programme outcome, recurring CMOC patterns were grouped into conceptual categories. Data analysis was led by KON and FR, with regular team discussions about CMOCs [with SMH, ER, and CP]. We developed separate CMOCs for the two models of care (routine management and management of the diabetic foot disease).

##### Outcome assessment

Though the data was predominantly qualitative, some outcomes were defined both qualitatively and quantitatively. For example, data were available to assess the implementation of certain components of the programme. First, integrated DNS routinely collect data on their activity, including GP practice visits, patient episodes of care, and service capacity [47]. DNS activity was analysed and reported per 1 Whole Time Equivalent (WTE) and across defined areas of community healthcare services outside of acute hospitals, called Community Healthcare Organisations (CHO). Second, a national survey was carried out with PNs (October 2016–May 2017) to examine their role in diabetes care. This 59-item survey included questions on how diabetes care is delivered at their practice, the current support in diabetes care available at the practice, including access to specialist services, and how they used these services. Nurses (n = 1466) were sent the survey via Professional Development Coordinators for Practice Nursing and the Irish Practice Nurse Association. Activity data and survey data were used to examine engagement with (i.e., whether the programme was adopted in primary care), and implementation of, the programme components. Descriptive statistics (N (%)) were generated for relevant questions.

##### Stage 4: Synthesis and interpretation

Data were then used to support, refine, or refute the initial CMOCs developed from stage 1 of the study. Similarities and overlaps between the CMOCs for the models of care were identified (by FR and KON) and presented to the wider research team (SMH, ER, CP). The group refined interpretations by explicitly seeking conflicting data and making comparisons across different contexts. Finally, CMOCs were consolidated to provide an explanation of why the programme worked for some but not others. Memos were used and shared throughout the analysis to note assumptions, coding definitions, hunches, and early impressions. The language and expressions of the participants were maintained as far as possible, using in vivo codes, to avoid losing the meaning and context.

The study was approved by the Clinical Research Ethics Committee of the Cork Teaching Hospitals.

## Results

Participants included 19 stakeholders purposively sampled from the NDP working group (Stage 1) and 38 professionals purposively sampled to represent different disciplines implementing the NDP in each case (Stage 2). More details of participants are provided in Additional File 3. In this section we first outline how the programme was expected to work based on documentary analysis and interviews with national stakeholders (Stage 1). This is followed by a series of refined theories (CMOCs) based on data collection during programme implementation, outlining the important contexts that influenced whether mechanisms produced intended and unintended outcomes (Stages 2–4).

### Formulating initial programme theories: How was the programme expected to work (Stage 1)

There was no formal or standardised dissemination route for the programme. It was assumed that information would ‘*filter down*’ (working group #3) through a variety of existing informal channels and networks. This included presentations to professional groups, largely comprising people already interested in diabetes quality improvement (e.g., training and conferences, existing connections to primary care initiatives, contact with a new integrated DNS or podiatrist, and local implementation groups). Due to this informal approach, there were varying levels of awareness about the programme among HCPs.

Those involved in the design of the models of care assumed that introducing protocols would standardise how care was provided, and that new clinical posts would become a catalyst for service change, facilitating integrated care at local level. It was expected that HCPs would buy in to new ways of working, see the value of new resources and, as a result, align their work practices accordingly to the new models of care. However, the changes outlined by the NDP were not mandated. Due to the socio-political and service context certain planned components of the programme did not materialise (Additional File 4), including the official release of the protocol for the model of integrated care for routine management. For those that did materialise, there was a disconnect between *design/planning* (i.e., the NDP working groups) and *implementation* once introduced into the health system (i.e., health service executive and primary care). Despite liaison structures in place between local hospitals, represented by the Diabetes Services Implementation Groups, the lines of accountability and responsibility were unclear once the programme was introduced.

“It’s not as clear cut as one crowd provide the policy and the other crowd will implement; there has to be a merging in the middle.” (working group #15)

### Refining initial programme theories: How the programme was implemented: in what contexts and by what mechanisms did it lead to (intended and unintended) outcomes (Stages 2–4)

Thus, in this context, where there was a disconnect between responsibility for *designing and planning* the programme and the responsibility for *implementation* once introduced into the health system, HCPs used their professional judgment to engage with, implement, and adapt the new models of care to varying degrees and in ways that matched their immediate priorities and circumstances (Table 1). This was not limited to one stakeholder group; GPs, nurses, specialists, and podiatrists made professional judgements about whether and how to engage with the programme resources. Details of the overarching CMOCs for the programme are provided in Table 1. Additional individual CMOCs supporting them along with exemplar quotes are available in Additional File 5.

**Table 1:** Summary of CMOs.


**Overarching CMOC** Varying levels of awareness about the programme (C), no planned approach to implementation (C), and professional oversight (C), and resource demands within the primary and secondary care health system (C) meant that it was more likely HCPs used their own professional judgment (M) to implement the models of care in ways that matched their priorities (O) with varying degrees of engagement (O) and implementation (O).	

**CMOC 1: Weighing up the advantage of engagement in primary care**	

Depending on their experience delivering diabetes care (C) and resource demands at each primary care practice (C), HCPs weighed up the relative advantage of the programme over their current approach to diabetes care (M) which led to variability in their willingness to engage with the programme (O).	

**CMOC 2: Legitimising a new role in secondary care**	

Given different levels of familiarity with the role of integrated DNS and podiatrists among existing HCPs (C), and depending on the availability of published protocols which provided guidance on the new roles (C), new HCPs recruited through the programme made judgements about how to legitimise their role (M) to engage HCPs with the programme and establish role boundaries (O).	

**CMOC 3: Implementation; adjusting to fulfil immediate local need**	

**3.1 Implementation in primary care** Depending on HCP’s resources (C) or experience in diabetes (C), they felt more or less supported by the programme (M) and therefore, adapted the programme components to meet their immediate needs (O).	**3.2 Implementation in secondary care** Depending on familiarity with the role of integrated DNS and podiatrists among existing HCPs (C), resources (C) and existing professional boundaries in secondary care (C), new HCPs recruited by the programme and secondary care HCPs made judgements about how to best adapt the role (M) to meet immediate service needs (O).


### Initial engagement in primary care

#### Weighing up relative advantage

Primary care HCPs’ experience providing diabetes care, and resource demands in the practice at that time, influenced whether they perceived the programme to be more or less advantageous than current care delivery (e.g., level of diabetes care they were providing already), and whether they saw it as an opportunity to receive more support or to improve care (e.g., a need for the programme resources) (see Additional File 5, CMO 1.4, 1.5). In some cases, recognising this immediate need for resources motivated HCPs to work around feasibility issues (e.g., practice space) (Additional File 5, CMO 1.6).

“Well, we saw the value of it [integrated DNS service] … we’ve a lot of people with diabetes and we’ve a lot of young diabetics and so to us, it just seemed like a good idea.” (PN#7)

Where practices had sufficient resources (e.g., access to specialists) and believed their care was good, they were unsure how the new programme would align with or improve current practice (Table 1, CMO 1). Some contexts were contingent on others with respect to implementation; for example, once an integrated DNS was embedded in the practice, practice nurses felt more supported, changing the context, and leading them to become more engaged in the management of diabetic foot disease (Additional File 5, CMO 1.5).

“We had the set-up. We probably hadn’t the protocol as such. We would do the full blood count, the A1C, the cholesterol and that, but we wouldn’t have weighed them on each visit, or done the BMI, nor would we have done the foot examination unless they had a problem…I’m trying now, a bit, since [integrated DNS] gave me all the literature, and I have the books.” (PN#15)

#### Resistance to engagement

In some practices, there were exceptions. Due to the breakdown of GP contract negotiations [48] and owing to the position of GPs as sole traders, the new programme was perceived by some as undermining practices and their resources. Interviewees suggested that the programme did not adequately reimburse the cost of implementation (Additional File 5, CMO 1.3).

“You can’t expect GPs to fork out and pay for facilities for nurses and build rooms for them and everything else…And become an, an outpost to the hospitals so they provide services.” (GP#4)

### Implementation in primary care: adjusting to need

In a similar way to how professionals initially engaged in the programme, *implementation* of the programme components in practice depended largely on HCPs’ resources or experience in diabetes. These two contexts identified in our qualitative data, appeared to explain the extent to which HCPs felt supported (or not) by the programme, and whether they were likely to adjust protocols and the new roles to fulfil their immediate needs. Depending on resources, practices referred different cases to the integrated DNS; some referred people with uncomplicated diabetes and others referred those considered more complex (Additional File 5, CMO 3.1.2). This variation was also evident in the activity data collected by integrated DNS (n = 29) (Additional File 6) [47] and survey data. Among practice nurses who were using the integrated DNS service (n = 215), 30% reported that the integrated DNS supported the practice by reviewing patients with uncomplicated type 2 diabetes. The intention was that practice nurses would upskill by observing the integrated DNS during clinics, however primary care HCPs judged that ‘*doubling up*’ (DNS#13) was not the best use of resources (Additional File 5, CMO 3.1.3).

“Some of the busier practices, no [the practice nurse does not sit in with me], not always, they will book the patients in but they cannot afford the practice nurse hours on a morning or an afternoon to be spent doing it.” (DNS#6)

### Legitimising the new roles in secondary care

Two factors played a part in whether the roles of newly recruited staff were implemented as intended in the secondary care setting: 1) the availability of guidance documents which afforded some legitimacy to, and 2) the level of familiarity with, the specialist role. Based on these factors, integrated DNS and podiatrists judged the extent to which they would promote and engage in negotiations about their role. Where secondary care staff were unfamiliar with the role of new HCPs, the HCPs were more uncertain about (the legitimacy of) their role within the team and whether their contribution to diabetes management would be accepted. This uncertainty led them to engage in efforts to increase awareness of their expertise among other HCPs (Table 1, CMO 2.1).

Integrated DNS and podiatrists differed in the way they engaged in negotiations about their role. The role of hospital DNS was already well-established and in the absence of formal guidance on this new ‘integrated’ role that would span primary and secondary care, conditions did not support integrated DNS to assert professional boundaries. Where integrated DNS were previously part of the hospital team, they were not necessarily willing to challenge the status quo (Additional File 5, CMO 2.2). This contrasted to podiatrists who had a published protocol and so were more comfortable asserting their role. They could rely on this document to assert their role in the team (Additional File 5, CMO 2.3).

“We needed something (guidance) to say this is what has to be done, you know, that’s what kind of made it easier going in implementing these changes, like “listen, it’s not me, it’s the programme, you have to do it”. (Podiatrist #12)

### Implementing new roles in secondary care: adjusting to service need

The implementation of new roles in secondary care depended on professional boundaries/working relationships and demands on the current service. In the case of podiatrists, the decision about how the role was implemented depended on whether the HCPs in secondary care were receptive to facilitating the new service. With support at consultant level and the presence of an established MDT, podiatrists felt empowered to implement the risk stratified pathways as intended, or adjust as required (Additional File 5, CMO 3.2.1).

“A lot of the [podiatrists] that I know wouldn’t have support from the diabetes consultant at podiatric level…[it has] taken almost a year to get it up and running. If I wasn’t attached to [the hospital], we wouldn’t have this (service)” (Podiatrist #14)

Similar to the response in primary care, HCPs in secondary care adapted the role to meet immediate service needs. In the case of the integrated DNS role, the decision about how the role was implemented depended on hospital resources, specifically staffing levels, and the demand for the service (Additional File 5, CMO 3.2.2). This influenced the type of patients (i.e., type 1 or 2) seen by the integrated DNS during their time in the hospital.

“So now what we end up doing is actually putting [the integrated DNS] into our clinics, to save one of our other nurses for the other patients, seeing as we’re short on nurses.” (Endocrinologist #2)

In the case of the podiatrists, demand on the service also influenced the implementation of the risk stratified pathways. The shortage of podiatry services prior to implementation and the securement of only 16 podiatry positions in secondary care meant that podiatry services were “*snowed under with work*” (working group #17) and “*stretched to the limit*” (working group #4). As a result, podiatrists aimed to provide the best service they could with the resources available (Additional File 5, CMO 3.2.3). Depending on the availability of community podiatry services, risk stratified pathways were only partially implemented with podiatrists only managing to address active or high-risk foot disease and not moderate risk as outlined in the model of care.

“Moderate risks have been neglected, for the last year, just because our caseload of high risk patients has just gone through the roof.” (Podiatrist #20)

## Discussion

Our aim was to understand how and why a national programme to introduce integrated care for routine management of diabetes and diabetic foot disease worked (or not), in different settings and for different groups. Unlike previous evaluations of integrated diabetes care, we used a realist approach to determine the mechanisms and the contextual factors that bring about change. Our results suggest that at the outset there was an implicit assumption that HCPs would buy into a new way of working which involved greater multidisplinarity, and delivery of care across professional boundaries. However, there was a disconnect between responsibility for *designing and planning* the programme and the responsibility for *implementation* once introduced into the health system. In this context, HCPs used their own judgment on whether to adopt, or adjust the programme which influenced how the programme worked on the ground.

### Implications

Our findings have three key implications which are relevant given broader reforms to support integrated care. First, the study highlights the importance of developing a shared awareness of integrated care programmes and their aims among *all* key actors/implementers. One of the programme aims was to involve different clinical disciplines to co-produce service change, i.e., involving professionals (and patients) in jointly designing and planning how the new service might look, therefore increasing ownership and commitment. However, despite this process at the *design* stage, at the *implementation* stage, primary care HCPs engaged with, and implemented the programme based on whether they saw a relative advantage. This is in line with existing research which suggests professional judgements and adaptations are often part of the work done to embed service changes [49], with adaptations often a response to organisational challenges such as staffing [20], clinical experience [39,50], working relationships [51] and internal or external rewards [39]. To support implementation of the programme, new staff had to champion their own role, negotiate their role boundaries, challenge the status quo, and serve as catalyst for integrated care, communicating information about service changes. However, their capacity to do so depended on others’ *familiarity* with the role and their relationships with existing staff, together with external validation and the legitimacy afforded by guidelines. It was challenging for individual HCPs to achieve this without complementary systematic service level communication, the lack of which was highlighted in a recent evaluation of the national clinical programmes as a whole [52], or changes to existing team structures in primary or secondary care [53]. International work suggests that when clinicians are working across boundaries – physical boundaries between care settings, or professional boundaries between disciplines within a multidisciplinary teams – there will be issues in terms of the understanding and implementation of new roles [54,55,56]. Previous studies of whole system change [57,58], have reported tensions between clinical professions, arising from the fact the programme challenged accepted role boundaries and the status quo. In their study of health services modernisation, Huby et al linked the momentum of service reform to the degree to which role boundaries could be negotiated [59]. The success of integrated care in England [60], Scotland [61], Belgium [62], Canada [63] and the Netherlands [10], has been linked to efforts to cultivate a shared culture and understanding of new roles and changes to existing roles [61], and whether or not there is a tradition and understanding of interdisciplinary work [10,60,62,63].

Second, it is important to strike a balance between standardisation and facilitating professional autonomy. In the current study, primary care HCPs asserted their autonomy when deciding to engage with and implement the programme. New staff were expected to assert their autonomy to drive implementation of integrated care and negotiate their role. While research with HCPs and service managers in the UK suggests striking this balance is key to successful implementation [64], there are some important considerations. HCPs may see standardisation as a challenge to their autonomy [10]. Also, while some flexibility may facilitate implementation into existing practice [49], this can create an acceptance of delivering new interventions differently than intended [an agreed standard or protocol], with a negative impact on effectiveness and the quality of patient care [65]. These issues further indicate the importance of generating a shared understanding of the what and why of new integrated care programmes.

Third, our results indicate the importance of the local service infrastructure given that standardisation of new care models depends on the ‘starting point’, that is, the existing standard of care, resources, and experience, all of which drove HCP judgements about implementation. The programme in Ireland, like other diabetes integrated care programmes [10,62], seeks to standardise care delivery to address continuing variation [66,67]. Yet, regional differences in resourcing in both secondary and primary care influenced programme implementation, issues which are relevant internationally [68,69,70,71,72,73,74]. These challenges meant there are few local structures in place to create opportunities for ‘integrative’ working, such as shared clinics between practice nurses and integrated DNS in primary care, or referral pathways between community and hospital podiatrists. The unintended consequences of a shortage of resources like integrated DNS and podiatrist posts was also evident in the current study; specifically, the loss of the prevention aspect for diabetic foot disease with moderate risk patients not seen as frequently as necessary.

### Recommendations

There are three broad recommendations for the implementation of integrated care arising from this evaluation. First, preparatory work is needed to raise awareness of and support health professionals in new roles to implement integrated care. Strategies include providing protocols which outline the role – new clinical responsibilities [75,76] – local ‘champions’ or opinion leaders [77,78] who are already embedded in the setting [62,76,79], or taking a more structured (e.g., unified ‘big bang’ [80]) approach to communicating the goals and benefits of the new models of care.

Second, the *core* components of new care models required for effectiveness and which elements are optional or can be modified to fit with practice needs should be clarified with frontline HCPs. Without clarity on the core components, the result may be to exacerbate existing variation and widen the gap between the care provided in different parts of the country. While co-production of service change at the design stage is important; it is not a once-off process, and ongoing co-production and local tailoring during the implementation phase is to be expected. Policymakers should not assume that service changes can be entirely prescriptive. Implementation should be supported in such a way that it leads to desired outcomes, for example, empowering HCPs and strengthening their capacity to adapt both their roles and new interventions.

Third, local service infrastructures which can support integrated care (i.e., staff or remuneration for staff time, training, space) should be developed. HCPs have competing priorities and will make decisions about implementation within constraints [81,82]. Policies at a national level need to account for the fact that efforts to standardise diabetes or chronic disease management take place in a context where there are ‘pockets of excellence’. With different starting points and circumstances, a ‘one size fits all’ approach to implementation may not be a realistic or optimal goal for everyone. On this point, we suggest implementation support may need to be tailored accordingly, to ensure the core components can be delivered across the board. For example, some practices may need more intensive support from an integrated DNS to build confidence and expertise, or, in the absence of practice space, the option to refer patients to an integrated DNS based at another location in the community such as a primary care health centre.

Our findings highlight the early challenges to implementation. The service and policy context has continued to change, which may contribute to a more supportive context for integrated care. The integrated DNS and hospital podiatrist roles have now been in place for over five years [47]. Those who are longer in the role are embedded in local services and may be able to provide support and act as champions for colleagues in newer posts. A GP contract has been negotiated and secured [82].

### Strengths and limitations

To our knowledge this is the first realist evaluation of the implementation of an integrated diabetes care programme. While several disease specific programmes have been introduced internationally [9,10,12,13,14,15,16,17,18,19,20], few have interrogated how they are implemented. Barriers and facilitators to implementation are often represented as unconnected lists operating at higher, macro-level [83]. We have shown here benefits to understanding micro-level decisions and negotiations by those receiving a new programme on the ground. However, our evaluation has limitations which should be mentioned.

It was difficult to define cases and draw boundaries around ‘local diabetes services’. Case study boundaries in this study were somewhat fuzzy because, in Ireland, patients are not restricted to hospitals or practices in a defined geographic area and practices may refer into multiple community specialists and hospitals, sometimes based on historical or local arrangements. This may be why we did not identify regional boundaries as an important context for implementation.

We did not have access to routine administrative data on processes of care that would reflect whether implementation was happening or not. Moreover, given those involved in the development of the programme were not explicit in terms of what fidelity to the programme looked like, it was challenging to assess the implementation of specific processes and procedures reflective of integrated care.

Professionals were purposively sampled to represent relevant stakeholders in an area, and theoretical sampling was used to follow lines of inquiry. However, this was not always successful. For example, we failed to recruit HCPs without an interest in diabetes quality improvement (an important contextual factor). This notwithstanding, we did identify some disconfirming cases; integrated DNS spoke about the challenging of engaging with some practices, and GPs who had adopted the programme, spoke about resistance to adoption among colleagues.

The realist analysis allowed an in depth understanding of implementation in different contexts, and in particular this approach facilitated us to generate transferable lessons about how models of integrated care work, rather than learning specific to a single context or intervention. This was a key strength as it ensures our findings are relevant for future implementation of models of integrated care and maintain a policy-relevance beyond the specific interventions studied. However, it was also extremely time consuming. This was largely because analysis done manually in Microsoft Word using an approach similar to that outlined by Jackson et al [84]. That is, we identified dyads (CO, MO, CM) and triads (CMO) within individual participant transcripts, formulating multiple CMOCs within one interview transcript. This enabled us to make logical inferences across transcripts. Immersion in data analysis was particularly important to be able to make these inferences however, in a practical sense dedicating longer blocks of time to data analysis was an ongoing challenge. While this work could be done independently for different models of care by different researchers (FR: routine management; KON: management of the diabetic foot disease), regular discussions with the wider team were crucial to formulate the final programme theory. These discussions were particularly valuable at two specific timepoints; when sharing our findings on the two models of care and discussing similarities, and when considering our individual CMOCs and how they consolidate to form an overarching programme theory. The broad scope of the intervention (encompassing two models of care) made the evaluation challenging. We found theory-driven interviews, whereby we presented initial theories for two models to participants, demanded more explanation by the researcher and could be lengthy, particularly for those HCPs who could comment on both models (GPs, PNs).

## Conclusion

In summary, the implementation of whole system service changes should be supported both by a comprehensive implementation plan incorporating a communication strategy to establish a shared understanding and ownership, guidance which clarifies the core versus flexible programme components, together with ‘bottom up’ efforts to create structures and arrangements which can facilitate implementation which is tailored to local needs and different ‘starting points’.

## Additional File

The additional file for this article can be found as follows:

10.5334/ijic.5815.s1 Additional Files. Additional Files 1 to 6.

## References

[1] Nolte E, Knai C, Saltman RB. (eds.) Assessing chronic disease management in European health systems: Concepts and approaches. Copenhagen, Denmark: European Observatory on Health Systems and Policies; 2014. 29019637

[2] Cash-Gibson L, Rosenmoller M. Project INTEGRATE – a common methodological approach to understand integrated health care in Europe. International Journal of Integrated Care. 2014; 14: e035. DOI: 10.5334/ijic.1980 25550690 PMC4276036

[3] Shaw S, Rosen R, Rumbold B. What is integrated care? London: Nuffield Trust; 2011.

[4] World Health Organisation (WHO). ROADMAP. Strengthening people-centred health systems in the WHO European Region. WHO; 2013.

[5] Goodwin N. How do you build programmes of integrated care? The need to broaden our conceptual and empirical understanding. International Journal of Integrated Care. 2013; 13: e040. DOI: 10.5334/ijic.1207 24179459 PMC3812348

[6] Busetto L, Luijkx K, Calciolari S, Gonzalez Ortiz LG, Vrijhoef HJM. Exploration of workforce changes in integrated chronic care: Findings from an interactive and emergent research design. PLoS One. 2017; 12(12): e0187468. DOI: 10.1371/journal.pone.0187468 29267288 PMC5739393

[7] Kahn R, Anderson JE. Improving Diabetes Care: The Model for Health Care Reform. Diabetes Care. 2009; 32(6): 1115–8. DOI: 10.2337/dc09-0184 19460917 PMC2681040

[8] World Health Organisation (WHO). Global Report on Diabetes. France: WHO Press; 2016.

[9] Fuchs S, Henschke C, Blümel M, Busse R. Disease Management Programs for Type 2 Diabetes in Germany: A Systematic Literature Review Evaluating Effectiveness. Deutsches Ärzteblatt International. 2014; 111(26): 453–63. DOI: 10.3238/arztebl.2014.0453 25019922 PMC4101529

[10] Busetto L, Luijkx K, Huizing A, Vrijhoef B. Implementation of integrated care for diabetes mellitus type 2 by two Dutch care groups: a case study. BMC Family Practice. 2015; 16(1): 105. DOI: 10.1186/s12875-015-0320-z 26292703 PMC4546228

[11] Wadmann S, Strandberg-Larsen M, Vrangboek K. Coordination between primary and secondary healthcare in Denmark and Sweden. International Journal of Integrated Care. 2009; 19(12). DOI: 10.5334/ijic.302 PMC2663705 19340328

[12] Donohoe ME, Fletton JA, Hook A, Powell R, Robinson I, Stead JW, et al. Improving foot care for people with diabetes mellitus – a randomized controlled trial of an integrated care approach. Diabetic Medicine. 2001; 17(8): 581–7. DOI: 10.1046/j.1464-5491.2000.00336.x 11073179

[13] Smith S, Bury G, O’Leary M, Shannon W, Tynan A, Staines A, et al. The North Dublin randomized controlled trial of structured diabetes shared care. Family Practice. 2004; 21(1): 39–45. DOI: 10.1093/fampra/cmh109 14760042

[14] Diabetes Integrated Care Evaluation Team. Integrated care for diabetes: clinical, psychosocial, and economic evaluation. Diabetes Integrated Care Evaluation Team. BMJ. 1994; 308(6938): 1208–12. DOI: 10.1136/bmj.308.6938.1208 8180540 PMC2540045

[15] Rothe U, Muller G, Schwarz PE, Seifert M, Kunath H, Koch R, et al. Evaluation of a diabetes management system based on practice guidelines, integrated care, and continuous quality management in a Federal State of Germany: a population-based approach to health care research. Diabetes Care. 2008; 31(5): 863–8. DOI: 10.2337/dc07-0858 18332161

[16] Borgermans L, Goderis G, Van Den Broeke C, Verbeke G, Carbonez A, Ivanova A, et al. Interdisciplinary diabetes care teams operating on the interface between primary and specialty care are associated with improved outcomes of care: findings from the Leuven Diabetes Project. BMC Health Services Research. 2009; 9(1): 1–15. DOI: 10.1186/1472-6963-9-179 19811624 PMC2762969

[17] Walsh JL, Harris BH, Roberts AW. Evaluation of a community diabetes initiative: Integrating diabetes care. Primary Care Diabetes. 2015; 9(3): 203–10. DOI: 10.1016/j.pcd.2014.10.003 25498988

[18] Johnson M, Goyder E. Changing roles, changing responsibilities and changing relationships: an exploration of the impact of a new model for delivering integrated diabetes care in general practice. Quality in Primary Care. 2005; 13(2): 85–90 6p.

[19] McRae IS, Butler JRG, Sibthorpe BM, Ruscoe W, Snow J, Rubiano D, et al. A cost effectiveness study of integrated care in health services delivery: a diabetes program in Australia. BMC Health Services Research. 2008; 8: 205. DOI: 10.1186/1472-6963-8-205 18834551 PMC2577097

[20] Foster M, Burridge L, Donald M, Zhang J, Jackson C. The work of local healthcare innovation: a qualitative study of GP-led integrated diabetes care in primary health care. BMC Health Services Research. 2016; 16: 11. DOI: 10.1186/s12913-016-1270-4 26769248 PMC4712472

[21] Burridge LH, Foster MM, Donald M, Zhang J, Russell AW, Jackson CL. Making sense of change: patients’ views of diabetes and GP-led integrated diabetes care. Health Expectations. 2015. DOI: 10.1111/hex.12331 PMC5055219 25565290

[22] Yeo SQ, Harris M, Majeed FA. Integrated care for diabetes—a Singapore approach. International Journal of Integrated Care. 2012; 12: e8. DOI: 10.5334/ijic.810 22977434 PMC3429145

[23] Health Service Executive. National Clinical Programme for Diabetes. Available from: http://www.hse.ie/eng/about/Who/clinical/natclinprog/diabetesprogramme/ [Accessed: 4th May 2022].

[24] Riordan F, McHugh SM, Murphy K, Barrett J, Kearney PM. The role of nurse specialists in the delivery of integrated diabetes care: a cross-sectional survey of diabetes nurse specialist services. BMJ Open. 2017; 7(8). DOI: 10.1136/bmjopen-2016-015049 PMC5724109 28801394

[25] Mc Hugh SM, O’Keeffe J, Fitzpatrick A, de Siun A, O’Mullane M, Perry I, et al. Diabetes care in Ireland: a survey of general practitioners. Primary Care Diabetes. 2009; 3(4): 225–31. DOI: 10.1016/j.pcd.2009.09.002 19837640

[26] Mc Hugh SM, O’Mullane M, Perry IJ, Bradley C. Barriers to, and facilitators in, introducing integrated diabetes care in Ireland: a qualitative study of views in general practice. BMJ Open. 2013; 3(8): e003217. DOI: 10.1136/bmjopen-2013-003217 PMC3753479 23959754

[27] Elissen AM, Steuten LM, Lemmens LC, Drewes HW, Lemmens KM, Meeuwissen JA, et al. Meta-analysis of the effectiveness of chronic care management for diabetes: investigating heterogeneity in outcomes. Journal of Evaluation in Clinical Practice. 2013; 19(5): 753–62. DOI: 10.1111/j.1365-2753.2012.01817.x 22372830

[28] Hoogeveen RC, Dorresteijn JA, Kriegsman DM, Valk GD. Complex interventions for preventing diabetic foot ulceration. The Cochrane Database of Systematic Reviews. 2015(8): Cd007610. DOI: 10.1002/14651858.CD007610.pub3 PMC8504983 26299991

[29] Baxter S, Johnson M, Chambers D, Sutton A, Goyder E, Booth A. Understanding new models of integrated care in developed countries: a systematic review. Southampton, UK: NIHR Journals Library; 2018. DOI: 10.3310/hsdr06290 30148581

[30] Martinez-Gonzalez NA, Berchtold P, Ullman K, Busato A, Egger M. Integrated care programmes for adults with chronic conditions: a meta-review. International Journal of Quality in Health Care. 2014; 26(5): 561–70. DOI: 10.1093/intqhc/mzu071 PMC4195469 25108537

[31] Busetto L, Luijkx KG, Elissen AMJ, Vrijhoef HJM. Context, mechanisms and outcomes of integrated care for diabetes mellitus type 2: a systematic review. BMC Health Services Research. 2016; 16(1): 1–14. DOI: 10.1186/s12913-015-1231-3 26772769 PMC4715325

[32] Bongaerts BW, Müssig K, Wens J, Lang C, Schwarz P, Roden M, Rathmann W. Effectiveness of chronic care models for the management of type 2 diabetes mellitus in Europe: a systematic review and meta-analysis. BMJ Open. 2017 Mar 20; 7(3): e013076. DOI: 10.1136/bmjopen-2016-013076 PMC5372084 28320788

[33] Renders CM, Valk GD, Griffin S, Wagner EH, Eijk JT, Assendelft WJ. Interventions to improve the management of diabetes mellitus in primary care, outpatient and community settings. The Cochrane Database of Systematic Reviews. 2001; 1: Cd001481. DOI: 10.1002/14651858.CD001481 PMC7045779 11279717

[34] Marchal B, van Belle S, van Olmen J, Hoerée T, Kegels G. Is realist evaluation keeping its promise? A review of published empirical studies in the field of health systems research. Evaluation. 2012; 18(2): 192–212. DOI: 10.1177/1356389012442444

[35] Walshe K, Freeman T. Effectiveness of quality improvement: learning from evaluations. Quality and Safety in Health Care. 2002; 11(1): 85. DOI: 10.1136/qhc.11.1.85 12078378 PMC1743560

[36] Chen HT. Theory-driven evaluation: Conceptual framework, application and advancement. In: Evaluation von Programmen und Projekten für eine demokratische Kultur. 2012; 17–40. DOI: 10.1007/978-3-531-19009-9_2

[37] Greenhalgh T, Humphrey T, Hughes J, MacFarlane F, Butler C, Pawson R. How do you modernize a health service? A realist evaluation of whole-scale transformation in London. Milbank Quarterly. 2009; 87. DOI: 10.1111/j.1468-0009.2009.00562.x PMC2881448 19523123

[38] Ranmuthugala G, Cunningham FC, Plumb JJ, Long J, Georgiou A, Westbrook JI, et al. A realist evaluation of the role of communities of practice in changing healthcare practice. Implementation Science. 2011; 6: 49. DOI: 10.1186/1748-5908-6-49 21600057 PMC3120719

[39] Rycroft-Malone J, Fontenla M, Bick D, Seers K. A realistic evaluation: the case of protocol-based care. Implementation Science. 2010; 5: 38. DOI: 10.1186/1748-5908-5-38 20504293 PMC2889857

[40] Pawson R, Tilley N. Realist evaluation, 2004. Available from: http://www.communitymatters.com.au/RE_chapter.pdf [Accessed: 5th May 2022].

[41] Mc Hugh S, Marsden P, Brennan C, Murphy K, Croarkin C, Moran J, et al. Counting on commitment; the quality of primary care-led diabetes management in a system with minimal incentives. BMC Health Services Research. 2011; 11: 348. DOI: 10.1186/1472-6963-11-348 22204759 PMC3315762

[42] Health Service Executive (HSE). Diabetes Expert Advisory Group. First Report: April 2008. Kildare: HSE; 2008.

[43] Health Service Executive (HSE). Model of Care for the Diabetic Foot. Dublin: HSE; 2011.

[44] McHugh SM, Tracey ML, Riordan F, O’Neill K, Mays N, Kearney PM. Evaluating the implementation of a national clinical programme for diabetes to standardise and improve services: a realist evaluation protocol. Implementation Science. 2016; 11(1): 107. DOI: 10.1186/s13012-016-0464-9 27464711 PMC4964144

[45] Wong G, Westhorp G, Manzano A, Greenhalgh J, Jagosh J, Greenhalgh T. RAMESES II reporting standards for realist evaluations. BMC Medicine. 2016; 14(1): 96. DOI: 10.1186/s12916-016-0643-1 27342217 PMC4920991

[46] Project. TRI. Retroduction in realist evaluation; 2017.

[47] Riordan F, McGrath N, McHugh SM, Kearney PM, Twamley H, Smyth N. Overview of Activity Data in Primary Care from Clinical Nurse Specialist (CNSp) Diabetes Integrated Care Group National Clinical Programme for Diabetes (NCPD); 2018. Available from: https://www.hse.ie/eng/about/who/cspd/ncps/diabetes/resources/an-overview-of-activity-data-from-clinical-nurse-specialist-report-rev-8–2019.pdf [Accessed 5th May 2022].

[48] College of GPs withdraws from HSE clinical programmes in protest over fees [press release]. The Irish Times (online); 2013. Available from: https://www.irishtimes.com/news/ireland/irish-news/college-of-gps-withdraws-from-hse-clinical-programmes-in-protest-over-fees-1.1466517. [Accessed 5th May 2022].

[49] Stirman SW, Miller CJ, Toder K, Calloway A. Development of a framework and coding system for modifications and adaptations of evidence-based interventions. Implementation Science. 2013; 8: 65. DOI: 10.1186/1748-5908-8-65 23758995 PMC3686699

[50] Hancock HC, Easen PR. The decision-making processes of nurses when extubating patients following cardiac surgery: an ethnographic study. International Journal of Nursing Studies. 2006; 43(6): 693–705. DOI: 10.1016/j.ijnurstu.2005.09.003 16256118

[51] Rycroft-Malone J, Fontenla M, Seers K, Bick D. Protocol-based care: the standardisation of decision-making? Journal of Clinical Nursing. 2009; 18(10): 1490–500. DOI: 10.1111/j.1365-2702.2008.02605.x 19413539

[52] Darker CD, Nicolson GH, Carroll A, Barry JM. The barriers and facilitators to the implementation of National Clinical Programmes in Ireland: using the MRC framework for process evaluations. BMC Health Services Research. 2018; 18(1): 733. DOI: 10.1186/s12913-018-3543-6 30249262 PMC6154419

[53] Riordan F, McGrath N, Dinneen SF, Kearney PM, McHugh SM. ‘Sink or Swim’: A Qualitative Study to Understand How and Why Nurses Adapt to Support the Implementation of Integrated Diabetes Care. International Journal of Integrated Care. 2019; 19(2): 2. DOI: 10.5334/ijic.4215 PMC6450245 30971868

[54] Martin GP, Currie G, Finn R. Reconfiguring or reproducing intra-professional boundaries? Specialist expertise, generalist knowledge and the ‘modernization’ of the medical workforce. Social Science & Medicine. 2009; 68(7): 1191–8. DOI: 10.1016/j.socscimed.2009.01.006 19201073

[55] Nancarrow SA, Borthwick AM. Dynamic professional boundaries in the healthcare workforce. Sociology of Health and Illness. 2005; 27(7): 897–919. DOI: 10.1111/j.1467-9566.2005.00463.x 16313522

[56] Rushforth B, McCrorie C, Glidewell L, Midgley E, Foy R. Barriers to effective management of type 2 diabetes in primary care: qualitative systematic review. British Journal of General Practice. 2016; 66(643): e114–27. DOI: 10.3399/bjgp16X683509 PMC4723210 26823263

[57] Hunter B. Implementing a national policy initiative to support normal birth: lessons from the All Wales Clinical Pathway for Normal Labour. Journal of Midwifery & Women’s Health. 2010; 55(3): 226–33. DOI: 10.1016/j.jmwh.2009.12.014 20434082

[58] Cheyne H, Abhyankar P, McCourt C. Empowering change: realist evaluation of a Scottish Government programme to support normal birth. Midwifery. 2013; 29(10): 1110–21. DOI: 10.1016/j.midw.2013.07.018 23968777

[59] Huby G, Harris FM, Powell AE, Kielman T, Sheikh A, Williams S, et al. Beyond professional boundaries: relationships and resources in health services’ modernisation in England and Wales. Sociology of Health and Illness. 2014; 36(3): 400–15. DOI: 10.1111/1467-9566.12067 24266800

[60] Ling T, Brereton L, Conklin A, Newbould J, Roland M. Barriers and facilitators to integrating care: experiences from the English Integrated Care Pilots. International journal of Integrated Care. 2012; 12: e129. DOI: 10.5334/ijic.982 23593044 PMC3601528

[61] Pearson C, Watson N. Implementing health and social care integration in Scotland: Renegotiating new partnerships in changing cultures of care. Health & Social Care in the Community. 2018; 26(3): e396–e403. DOI: 10.1111/hsc.12537 29349854

[62] Sunaert P, Bastiaens H, Feyen L, Snauwaert B, Nobels F, Wens J, et al. Implementation of a program for type 2 diabetes based on the Chronic Care Model in a hospital-centered health care system: “the Belgian experience”. BMC Health Services Research. 2009; 9(1): 152. DOI: 10.1186/1472-6963-9-152 19698185 PMC2757022

[63] Longpre C, Dubois CA. Fostering development of nursing practices to support integrated care when implementing integrated care pathways: what levers to use? BMC Health Services Research. 2017; 17(1): 790. DOI: 10.1186/s12913-017-2687-0 29187191 PMC5706162

[64] Lawton R, Parker D. Procedures and the professional: the case of the British NHS. Social Science & Medicine. 1999; 48(3): 353–61. DOI: 10.1016/S0277-9536(98)00345-1 10077283

[65] Barach P, Phelps G. Clinical sensemaking: a systematic approach to reduce the impact of normalised deviance in the medical profession. Journal of the Royal Society of Medicine. 2013; 106(10): 387–90. DOI: 10.1177/0141076813505045 24097963 PMC3791099

[66] Stone MA, Charpentier G, Doggen K, Kuss O, Lindblad U, Kellner C, et al. Quality of care of people with type 2 diabetes in eight European countries: findings from the Guideline Adherence to Enhance Care (GUIDANCE) study. Diabetes Care. 2013; 36(9): 2628–38. DOI: 10.2337/dc12-1759 23628621 PMC3747883

[67] Khunti K, Ceriello A, Cos X, De Block C. Achievement of guideline targets for blood pressure, lipid, and glycaemic control in type 2 diabetes: A meta-analysis. Diabetes Research and Clinical Practice. 2017; 137: 137–48. DOI: 10.1016/j.diabres.2017.12.004 29325774

[68] Barry MBK, Brick A, Morgenroth E, Normand C, O’Reilly J, Thomas S, Wiley M. Projecting the Impact of Demographic Change on the Demand for and Delivery of Health Care in Ireland. Dublin: Economic and Social Research Institute; 2009.

[69] McDaid DWM, Maresso M, Mossialos E. Ireland: Health system review. Health Systems in Transition. European Observatory on Health Systems and Policies; 2009.

[70] National Doctor Training and Planning HD. Medical Workforce Planning. Future Demand for General Practitioners. 2015–2025. Dublin: Health Service Executive; 2015.

[71] Majeed A. Shortage of general practitioners in the NHS. BMJ. 2017; 358: j3191. DOI: 10.1136/bmj.j3191 28694250

[72] Papp M, Korosi L, Sandor J, Nagy C, Juhasz A, Adany R. Workforce crisis in primary healthcare worldwide: Hungarian example in a longitudinal follow-up study. BMJ Open. 2019; 9(7): e024957. DOI: 10.1136/bmjopen-2018-024957 PMC6661691 31340955

[73] Berwick DM, Hackbarth AD. Eliminating Waste in US Health Care. JAMA. 2012; 307(14): 1513–6. DOI: 10.1001/jama.2012.362 22419800

[74] Petterson SM, Liaw WR, Tran C, Bazemore AW. Estimating the residency expansion required to avoid projected primary care physician shortages by 2035. Annals of Family Medicine. 2015; 13(2): 107–14. DOI: 10.1370/afm.1760 25755031 PMC4369588

[75] Bryant-Lukosius D, Dicenso A, Browne G, Pinelli J. Advanced practice nursing roles: development, implementation and evaluation. Journal of Advanced Nursing. 2004; 48(5): 519–29. DOI: 10.1111/j.1365-2648.2004.03234.x 15533090

[76] Contandriopoulos D, Brousselle A, Dubois C-A, Perroux M, Beaulieu M-D, Brault I, et al. A process-based framework to guide nurse practitioners integration into primary healthcare teams: results from a logic analysis. BMC Health Services Research. 2015; 15: 78. DOI: 10.1186/s12913-015-0731-5 25889415 PMC4349481

[77] Powell BJ, Waltz TJ, Chinman MJ, Damschroder LJ, Smith JL, Matthieu MM, et al. A refined compilation of implementation strategies: results from the Expert Recommendations for Implementing Change (ERIC) project. Implementation Science. 2015; 10: 21. DOI: 10.1186/s13012-015-0209-1 25889199 PMC4328074

[78] Flodgren G, Parmelli E, Doumit G, Gattellari M, O’Brien MA, Grimshaw J, et al. Local opinion leaders: effects on professional practice and health care outcomes. Cochrane Database of Systematic Reviews. 2011; 8. DOI: 10.1002/14651858.CD000125.pub4 PMC4172331 21833939

[79] Willard C, Luker K. Working with the team: strategies employed by hospital cancer nurse specialists to implement their role. Journal of Clinical Nursing. 2007; 16(4): 716–24. DOI: 10.1111/j.1365-2702.2006.01560.x 17402953

[80] Fulop NJ, Ramsay AI, Perry C, Boaden RJ, McKevitt C, Rudd AG, et al. Explaining outcomes in major system change: a qualitative study of implementing centralised acute stroke services in two large metropolitan regions in England. Implementation Science. 2016; 11(1): 80. DOI: 10.1186/s13012-016-0445-z 27255558 PMC4891887

[81] Djulbegovic B, Elqayam S. Many faces of rationality: Implications of the great rationality debate for clinical decision-making. Journal of Evaluation in Clinical Practice. 2017; 23(5): 915–22. DOI: 10.1111/jep.12788 28730671 PMC5655784

[82] Health Service Executive. Chronic Disease Management Programme; 2020. Available from: https://www.hse.ie/eng/about/who/gmscontracts/2019agreement/chronic-disease-management-programme/ [Accessed 5th May 2022].

[83] Suter E, Oelke ND, Adair CE, Armitage GD. Ten Key Principles for Successful Health Systems Integration. Healthcare Quarterly (Toronto, Ontario). 2009; 13(Spec No): 16–23. DOI: 10.12927/hcq.2009.21092 PMC3004930 20057244

[84] Jackson SF, Kolla G. A New Realistic Evaluation Analysis Method: Linked Coding of Context, Mechanism, and Outcome Relationships. American Journal of Evaluation. 2012; 33(3): 339–349. DOI: 10.1177/1098214012440030

