# Supplementary material for: Adoption and Initial Implementation of a National Integrated Care Programme for Diabetes: A Realist Evaluation
Source: Int J Integr Care. 2022 Jul 14;22(3):3. doi: 10.5334/ijic.5815 (PMC9284993; doi:10.5334/ijic.5815)
Supplement: Additional Files. — Additional Files 1 to 6. [file ijic-22-3-5815-s1.zip › s1-ijic-5815_riordan/file1-ijic-5815_riordan.docx]

| **Table 1** Role of hospital and integrated DNS | |
| --- | --- |
| **Hospital DNS** | **Integrated DNS** |
| 100% WTE in hospital | 80% WTE in primary care and 20% in secondary care |
| Secondary care  Provide care to adult patients with type 1 and complicated type 2 diabetes   - Deliver in-service professional education - Initiate and participate in evaluations and audits | Secondary care   - Provide care to adult patients with type 1 and complicated type 2 diabetes - Case management liaison with the consultant endocrinologist and MDT for patients reviewed in primary care - Assist with the development of integrated care pathways with hospital and primary care colleagues |
|  | Primary care   - Deliver clinics in GP practices - Deliver joint clinics with the practice nurse to build skills and confidence in the management of patients with uncomplicated type 2 diabetes - Review patients with complicated type 2 diabetes referred to them by the GP/nurse - Provide in-service education and training for all health care professionals - Discuss individual patient case management issues with the GP and nurse - Provide phone and email support to GP practices - Assist and support audit of diabetes care |

Abbreviations: DNS, Diabetes Nurse Specialist; WTE, Whole Time Equivalent; MDT, Multidisciplinary team
