# Supplementary material for: Adoption and Initial Implementation of a National Integrated Care Programme for Diabetes: A Realist Evaluation
Source: Int J Integr Care. 2022 Jul 14;22(3):3. doi: 10.5334/ijic.5815 (PMC9284993; doi:10.5334/ijic.5815)
Supplement: Additional Files. — Additional Files 1 to 6. [file ijic-22-3-5815-s1.zip › s1-ijic-5815_riordan/file1-ijic-5815_riordan.pdf]

Table 1 Role of hospital and integrated DNS

**Hospital DNS**	**Integrated DNS**
100% WTE in hospital	80% WTE in primary care and 20% in secondary care
Secondary care<br>Provide care to adult patients with type 1 and complicated type 2 diabetes<br>• Deliver in-service professional education<br>• Initiate and participate in evaluations and audits	Secondary care<br>• Provide care to adult patients with type 1 and complicated type 2 diabetes<br>• Case management liaison with the consultant endocrinologist and MDT for patients reviewed in primary care<br>• Assist with the development of integrated care pathways with hospital and primary care colleagues<br>Primary care<br>• Deliver clinics in GP practices<br>• Deliver joint clinics with the practice nurse to build skills and confidence in the management of patients with uncomplicated type 2 diabetes<br>• Review patients with complicated type 2 diabetes referred to them by the GP/nurse<br>• Provide in-service education and training for all health care professionals<br>• Discuss individual patient case management issues with the GP and nurse<br>• Provide phone and email support to GP practices<br>• Assist and support audit of diabetes care

Abbreviations: DNS, Diabetes Nurse Specialist; WTE, Whole Time Equivalent; MDT, Multidisciplinary team
