# Supplementary material for: Adoption and Initial Implementation of a National Integrated Care Programme for Diabetes: A Realist Evaluation
Source: Int J Integr Care. 2022 Jul 14;22(3):3. doi: 10.5334/ijic.5815 (PMC9284993; doi:10.5334/ijic.5815)
Supplement: Additional Files. — Additional Files 1 to 6. [file ijic-22-3-5815-s1.zip › s1-ijic-5815_riordan/file2-ijic-5815_riordan.docx]

| **Table 2** RAMESES II reporting standards | **Inclusion or justification for exclusion.** |
| --- | --- |
| **Item 1: Title**  In the title, identify the document as a realist evaluation. | Page 1 |
| Item 2: Summary or abstract | Page 2 |
| Introduction |  |
| Item 3: Rationale for evaluation Explain the purpose of the evaluation and the implications for its focus and design | Page 3, 4 |
| Item 4: Programme theory Describe the initial programme theory (or theories) that underpin the programme, policy or initiative. | Page 10 – described as part of the methods |
| Item 5: Evaluation questions, objectives and focus State the evaluation question(s) and specify the objectives for the evaluation. Describe whether and how the programme theory was used to define the scope and focus of the evaluation. | Page 5 |
| Item 6: Ethical approval State whether the realist evaluation required and has gained ethical approval from the relevant authorities, providing details as appropriate. If ethical approval was deemed unnecessary, explain why. | Page 10 – described as part of the methods |
| **Methods** |  |
| Item 7: Rationale for using realist evaluation Explain why a realist evaluation approach was chosen and (if relevant) adapted. | Page 4 -described as part of introduction |
| Item 8: Environment surrounding the evaluation Describe the environment in which the evaluation took place. | Page 5 – details of the study context |
| Item 9: Describe the programme, policy, initiative or product evaluated Provide relevant details on the programme, policy or initiative evaluated. | Page 5 – details of the national programme and Figure 1 showing the main components |
| Item 10: Describe and justify the evaluation design A description and justification of the evaluation design (i.e. the account of what was planned, done and why) should be included, at least in summary form or as an appendix, in the document which presents the main findings. If this is not done, the omission should be justified and a reference or link to the evaluation design given. It may also be useful to publish or make freely available (e.g. online on a website) any original evaluation design document or protocol, where they exist. | Page 6 and further detail in the study protocol (page 4) |
| Item 11: Data collection methods Describe and justify the data collection methods – which ones were used, why and how they fed into developing, supporting, refuting or refining programme theory. | Page 7, 8 |
| Item 12: Recruitment process and sampling strategy Describe how respondents to the evaluation were recruited or engaged and how the sample contributed to the development, support, refutation or refinement of programme theory. | Page 7, 8 |
| Item 13: Data analysis Describe in detail how data were analysed. This section should include information on the constructs that were identified, the process of analysis, how the programme theory was further developed, supported, refuted and refined, and (where relevant) how analysis changed as the evaluation unfolded. | Page 8, 9, 10 |
| Results |  |
| Item 14: Details of participants Report (if applicable) who took part in the evaluation, the details of the data they provided and how the data was used to develop, support, refute or refine programme theory. | Suppl. File 3 |
| Item 15: Main findings Present the key findings, linking them to CMO configurations. Show how they were used to further develop, test or refine the programme theory. | Page 10-18 |
| Discussion |  |
| Item 16: Summary of findings Summarise the main findings with attention to the evaluation questions, purpose of the evaluation, programme theory and intended audience. | Page 18 |
| Item 17: Strengths, limitations and future directions Discuss both the strengths of the evaluation and its limitations. These should include (but need not be limited to): (1) consideration of all the steps in the evaluation processes and (2) comment on the adequacy, trustworthiness and value of the explanatory insights which emerged. | Page 23 |
| Item 18: Comparison with existing literature Where appropriate, compare and contrast the evaluation’s findings with the existing literature on similar programmes, policies or initiatives. | Page 18-22 |
| Item 19: Conclusion and recommendations List the main conclusions that are justified by the analyses of the data. If appropriate, offer recommendations consistent with a realist approach. | Recommendations – page 22  Conclusions – page 24 |
| Item 20: Funding and conflict of interest State the funding source (if any) for the evaluation, the role played by the funder (if any) and any conflicts of interests of the evaluators. | Page 23 |
