# Supplementary material for: Adoption and Initial Implementation of a National Integrated Care Programme for Diabetes: A Realist Evaluation
Source: Int J Integr Care. 2022 Jul 14;22(3):3. doi: 10.5334/ijic.5815 (PMC9284993; doi:10.5334/ijic.5815)
Supplement: Additional Files. — Additional Files 1 to 6. [file ijic-22-3-5815-s1.zip › s1-ijic-5815_riordan/file3-ijic-5815_riordan.docx]

| **Table 3** Participants | | | | | |
| --- | --- | --- | --- | --- | --- |
|  | **Region** | | | |  |
| **Professional** | **R1** | **R2** | **R3** | **R4** | **Total** |
| Endocrinologist | 1 | 1 | 0 | 1 | 3 |
| Podiatrist | 2 | 2 | 1 | 1 | 6 |
| GP | 3 | 2 | 2 | 1 | 8 |
| Practice nurse | 3 | 2 | 2 | 3 | 10 |
| Diabetes Nurse Specialist | 3 | 2 | 2 | 4 | 11 |
|  |  |  |  |  |  |
| Total | 12 | 9 | 7 | 10 | 38 |
