# Supplementary material for: Adoption and Initial Implementation of a National Integrated Care Programme for Diabetes: A Realist Evaluation
Source: Int J Integr Care. 2022 Jul 14;22(3):3. doi: 10.5334/ijic.5815 (PMC9284993; doi:10.5334/ijic.5815)
Supplement: Additional Files. — Additional Files 1 to 6. [file ijic-22-3-5815-s1.zip › s1-ijic-5815_riordan/file3-ijic-5815_riordan.pdf]

**Table 3** Participants					
	**Region**				
**Professional**	**R1**	**R2**	**R3**	**R4**	**Total**
Endocrinologist	1	1	0	1	3
Podiatrist	2	2	1	1	6
GP	3	2	2	1	8
Practice nurse	3	2	2	3	10
Diabetes Nurse Specialist	3	2	2	4	11
Total	12	9	7	10	38
