# Supplementary material for: Adoption and Initial Implementation of a National Integrated Care Programme for Diabetes: A Realist Evaluation
Source: Int J Integr Care. 2022 Jul 14;22(3):3. doi: 10.5334/ijic.5815 (PMC9284993; doi:10.5334/ijic.5815)
Supplement: Additional Files. — Additional Files 1 to 6. [file ijic-22-3-5815-s1.zip › s1-ijic-5815_riordan/file4-ijic-5815_riordan.docx]

| **Table 4** Planned national clinical programme components and whether or not they were achieved | |
| --- | --- |
| **National Clinical Programme** | **Achieved** |
| **Integrated care model** |  |
| **Protocol** for risk stratified referral pathways. A contract to remunerate GPs for management of chronic disease in primary care was intended to be in place to support official sign of the guidance. | 🗶✓  Not officially signed off but version published in 2016 [52]  🗶 Negotiations failed, official GP withdrawal from clinical programmes [53] |
| **New staff:** Diabetes nurse specialists (80% community; 20% hospital) | ✓ but only available in some areas to some GPs |
| **Footcare model** |  |
| **Protocol** ‘National Model of Care for the Diabetic Foot’; risk stratified referral pathways for the diabetic foot | ✓ |
| **New staff**: podiatrists (100% hospital) | 🗶✓ but less than expected.  Due to the economic recession only 16 were introduced; not number required |
