# Supplementary material for: Adoption and Initial Implementation of a National Integrated Care Programme for Diabetes: A Realist Evaluation
Source: Int J Integr Care. 2022 Jul 14;22(3):3. doi: 10.5334/ijic.5815 (PMC9284993; doi:10.5334/ijic.5815)
Supplement: Additional Files. — Additional Files 1 to 6. [file ijic-22-3-5815-s1.zip › s1-ijic-5815_riordan/file4-ijic-5815_riordan.pdf]

Table 4 Planned national clinical programme components and whether or not they were achieved

National Clinical Programme	Achieved
**Integrated care model**	
**Protocol** for risk stratified referral pathways. A contract to remunerate GPs for management of chronic disease in primary care was intended to be in place to support official sign of the guidance.	✗✓ Not officially signed off but version published in 2016 [52]<br>✗ Negotiations failed, official GP withdrawal from clinical programmes [53]
**New staff:** Diabetes nurse specialists (80% community; 20% hospital)	✓ but only available in some areas to some GPs
**Footcare model**	
**Protocol** 'National Model of Care for the Diabetic Foot'; risk stratified referral pathways for the diabetic foot	✓
**New staff**: podiatrists (100% hospital)	✗✓ but less than expected. Due to the economic recession only 16 were introduced; not number required
