# Supplementary material for: Adoption and Initial Implementation of a National Integrated Care Programme for Diabetes: A Realist Evaluation
Source: Int J Integr Care. 2022 Jul 14;22(3):3. doi: 10.5334/ijic.5815 (PMC9284993; doi:10.5334/ijic.5815)
Supplement: Additional Files. — Additional Files 1 to 6. [file ijic-22-3-5815-s1.zip › s1-ijic-5815_riordan/file5-ijic-5815_riordan.pdf]

**Table 5** Context-Mechanism-Outcome configurations		
**#**	**CMO**	**Supporting quotes**
	**<u>Overarching CMO</u>**<br>Varying levels of awareness about the programme (C), no established approach to implementation (C), and professional oversight (C), and resource demands within the primary and secondary care health system (C) meant that it was more likely HCPs used their own professional judgment (M) to implement the programme in ways that matched their priorities (O) with varying degrees of engagement (O) with, and implementation of (O), the programme.	
1	**<u>Weighing up the advantage of engagement in primary care</u>**<br>Depending on their experience delivering diabetes care (C) and resource demands at each primary care practice (C), HCPs weighed up the relative advantage of the programme over current care delivery differently (M) which led to variability in their willingness to engage with the programme (O).	
1.1	*Confirmation bias*<br>Some practices perceived the care they deliver to be adequate for patient needs because they were already part of different structured diabetes care programmes (C), and did not see any relative advantage in the new programme (M) and were less willing to adopt the programme (O) and/or lacked awareness of how the intervention aligned with usual care at the practice (O).	*I am vaguely familiar with it (national clinical programme). I'm probably more familiar with the diabetic Structured Care programme, which we've been participating in for a while, and I understand that it's being rolled out almost nationally. Yes, the nurses have been checking peoples' feet for sensational pulses now for a long time and referring people to the chiropodist. Now, I think that's system's working, because it's not brought to my attention that it's not. (GP#7, R3)**

		They (ICN) don't come to the surgery here because I do the diabetic care. I have my diploma in diabetes...I go to loads of lectures about the diabetic foot ulcer. I've seen some awful pictures. I hope I never have anybody that bad in the surgery. I am glad to say that in all the years I've been doing this since 1998 we haven't... I don't put down classified (risk stratification). I haven't done that. I know them. We mind their feet. I've only had one diabetic ulcer here for all of the patients we have. The reason why she got it, she never came in. Poor attendee. I don't have any foot ulcers on any patients that have diabetes. I do not. (PN#9, R3)
1.2	*Feeling supported*<br>In some areas practices felt the care they currently deliver was adequate for patient needs because their patient cohort is not complex and/or they have good access to hospital (C), and therefore they already feel supported (M) did not perceive any relative advantage in the programme (M) and are less willing to adopt the programme (O)	*We haven't needed to [take on the DNS] because most of our patients are uncomplicated.….I think we're perfectly able to look after uncomplicated patients. I'm happy enough with that. It does work here, we work well. (PN#14, R1)*
1.3	*Resistance*<br>In the context of contract negotiations (poor relations) and the current business model of primary care (sole traders) (C), some GPs may have felt threatened by the programme (M) and believed that taking on the resource is not viable (O), the programme is not taking account of their needs (O) and responded with hostility/resist the programme (O).	*You can't expect GPs to, to fork out and pay for facilities for nurses and build rooms for them and everything else…And become an, an outpost to the hospitals so they provide services, nobody else in the state is expected to do that, it's ridiculous that they would expect general practice to do that. (GP#4, R1)*

		Some GPs who said they would not engage because financially it didn't make sense for them to be looking after chronic disease such as diabetes. (DNS#6, R4)<br><br>*The nurse in a general practice setting is a private employee and in the absence of remuneration, why should I pay my nurse to go on a course that the HSE want her to go on for, in a one single-handed practice might be a relatively small number of patients? So it did run into industrial relations issues. (Working group #13)*<br><br>*And this is just a personal example: so, when we got our podiatrists in X when there were podiatrists assigned I wrote to the GPs saying that we have podiatrists and they were for ... if you want, basically. And the IMO were on to me, a few GPs wrote me letters that were clearly written by the IMO but with their signature on it saying you know, 'Feck off' basically, 'we're not sending patients in to the podiatrists, we don't have to look after these patients'. 'Cop yourselves on', kind of thing. ((Laughs but clearly making a serious point)). So there's obviously clearly a heavily unionised group of GPs who are not happy with the proposals basically. Even though my letter was only telling them if they wanted to they could access the services for their patients. So, that's a major issue. I don't personally see it being resolved. (Working group #11)*

		I've had it directly from .. at conferences talking to GPs where they said that they were going to slam the door in the nurses face was the picture they were painting; that none of these nurses were going to be allowed work in a primary care setting. (Working group #1)<br><br>*Now, in some areas if there's very militant GPs where they don't want to be complying with the HSE on anything .. In fairness to the GPs, you have to understand, they're not salaried, they're private sole traders working out .. they've all their overhead costs, they've all their .. whatever (Working group #15)*
1.4	*Seeing an opportunity to improve care*<br>Some practices felt the care they currently deliver is not adequate because they have a particularly complex or large patient cohort and/or limited hospital access and/or short appointment slots (C); they saw the programme (and resources provided as part of it; integrated DNS) as an opportunity to improve (M) and therefore were likely to be willing to adopt the programme (O) and gain confidence and additional learning (O)	*Well, we saw the value of it and we saw… we've a lot of diabetics and we've a lot of young diabetics and so to us, it just seemed like a good idea (PN#7, R2)*<br><br>*We decided to take it on…. The general run of the diabetics I think we can manage fairly well, but there's always a few difficult ones that you're not sure if it's us or them (PN#15, R3)*<br><br>*She would have a better link with the consultants in there, so it's just a better care. (PN#8, R4)*<br><br>*We've learnt so much from them and it has given confidence in trying out the newer… because you're always worried about trying out new*

		treatments, new treatment options (PN#2, R2)<br><br>*GPs here are very open to you know, people coming in doing clinics and they're, you know, they're really open to that, it's much better care…It's time pressures, you see, it's going to, if they have somebody like that coming in, it's going to improve the care of the patients no end. (PN#15, R1)*<br><br>*It seemed like a no brainer. I mean, they're offering you specialist nurses. They're… it's like getting extra staff on for an hour or two or three, you know. It works. The patients like it. It really does make a difference. (PN#7, R2)*<br><br>*If I was free, I wouldn't mind sitting in on one or two of them [clinics]. It might give me a bit of help to maybe do better, you know? (PN#15, R3)*
1.5	*Seeing an opportunity to improve footcare*<br>Where practices provided some level of diabetes foot care and viewed it as 'part of the package' or had completed the foot training or linked in with the integrated DNS (C), practice nurses saw the new resource as an opportunity to improve (M) as it offered guidance and structure, and they felt confident to adopt their role in	*We've had a diabetic clinic here for a long time. So then obviously they were delighted then when it started off because then it gives more guidance and you know exactly what-have-you and we are all singing off the same hymn sheet, as it were...Well the foot screening service we do here is grand... It was but not probably to the same extent. We wouldn't*

	screening (O).	*have been probably as fussy or…It has changed, yes, because you obviously have the foot care there now to…We use the foot screening tool now...I think because it's part of the package. It's all part of diabetes, the same as everything else. (PN#2, R2)*<br><br>*Yeah, no, the training is important and the time… I mean, if you have a busy practice, you have a room full of people, and having seen the patient and you know patients, they're going to ask you ten other things while you're there and then you're going to say, 'Well, will you take off your shoes.' And again, they take them off and get them back on. It is time constraining. It really is. So you kind of learn to… while you're doing the papers, say, 'Well, you could be getting off your shoes there while I'm… but it's getting the confidence to be forward enough to say… to kind of push them along. (PN#7, R2)*<br><br>*We had the set-up. We probably hadn't the protocol as such. We would do the full blood count, the A1C, the cholesterol and that, but we wouldn't have weighed them on each visit, or done the BMI, nor would we have done the foot examination unless they had a problem. No. I'm trying now, a bit, since [DNS] gave me all the literature, and I have the books. I have the books for pre-diabetes and for newly diagnosed type 2 diabetes, and I*

		have the handouts now and I think that's a help...But time, really, is the biggest constraint...Then if they're slow to try and get off the shoe and sock... you do need time. It's nothing we can't do if we had more time to do it, really...Definitely it's [foot screening tool] sort of reignited me, anyway, to tune into it. (PN#15, R3)<br><br>*So, we would fill in the, kind of, form for podiatry care, or whatever. But it seems to be a bit of a lottery as to whether or not people get it, you know? …And there just seems to be several other criteria that, kind of are...a bit grey, to me certainly, it seems a bit confusing, you know...In terms of how to do a foot screening. But she's not confident anyway, so if she has any queries, she'll always run it by me, do you know what I mean? I: Yeah, because I know they're providing training for the Practice Nurses... R: Okay, well that would be good if it was available in the area. Hmm. (GP#6, R2)*
1.6	*Feasibility & workarounds*<br>In practices where they recognised the need for additional support because of patient complexity and cohort size (C), there may have been more willingness to find space and time workarounds to accommodate the programme (O), due to perceived urgency (M).	*You have to have space for them… Space is... well, that's… today was a nightmare... But we've managed to get the rooms on a different day….now, in our other surgery, we would be tight for rooms, but what we do is we open before the main surgery. We open at two instead of half three on the diabetic days (PN#7, R2)*

2	**Legitimising the new role in secondary care**<br>Given different levels of familiarity with the role of integrated DNS and podiatrists among existing HCPs (C1), and availability of published protocols which provided guidance on the new roles (C2), new HCPs recruited through the programme made judgements about how to legitimise their role (M) to engage HCPs with the programme and establish role boundaries (O).	
2.1	*Legitimising the role*<br>In contexts where staff were unfamiliar with the role of integrated DNS and podiatrists (C), new HCPs were more likely to feel uncertain about (the legitimacy of) their role and whether they would be accepted (M), leading them to feel they have to engage in efforts to increase awareness of their expertise and educate other practitioners in order to fulfil the aims of the intervention (O).	*I do think at that start but that then came down to her [DNS] educating people about her role and how the model of care should work, that this has now become… she is where she is now but again it has taken education again. (DNS#5, R3)*<br><br>*I want to push this role. I'm very clear on what my role is as an integrated care nurse. I work very well with structure. So, there's a list of what the integrated care nurse is supposed to be doing, and I have that in my mind all of the time. (DNS#4, R4)*<br><br>*I think it's one of those things, you're sort of going out there and pushing yourself and showing that this isn't just what we do, and sort of trying, going to meetings with them, shadowing them, that sort of thing. Get you nearer them and show what we can do. (Pod#10, R2)*

		A lot of people probably aren't aware what the podiatry profession is. So I think the podiatry profession has developed quite a lot over the last couple of years, and it's just, on our side of things, trying to get the word out there that we are able to deal with these patients. (Pod#20, R1)<br><br>*GPs – a lot of it as well was lack of knowledge and not knowing what we do. A lot of people don't even, like, a lot of GPs don't know what podiatry is. So it's just again, educating. Getting them to know what we do and where we are. (Pod#12, R2)*
2.2	*Being part of the team*<br>Where the integrated DNS already have existing working relationships (embeddedness) within the team (C), new HCPs introduced by the programme do not feel comfortable to assert their new role due to fear of upsetting the status quo (M) and make judgments about how to engage in professional negotiations around their role (O) leading some of them to adopt same role as other DNS which does not fully align with the intention of the new role (O)<br><br>Where the integrated DNS is new to the hospital (C) and there are no existing working relationships (C), they may feel more comfortable to assert their role (M) and engage in professional negotiations around their role (O).	*I suppose it was easier in some ways for me because I had worked in the hospital, in the same hospital that I'm going to now for the last number of years, so it was easier then to work a role out that suited everybody. (Pod#12, R2)*<br><br>*To be honest because I already worked in the [] hospital, I tend to go in and do my day like I was there other day of the week but generally what I do is when I go in, in the morning in [] hospital- it's the general nursing clinic so I just do what everybody, what the rest of the diabetes nurses do and it would have been what I did when I worked there before (DNS#13, R1)*

2.3	*Ownership*<br>Where the podiatrist has formal guidance on their new role and on the new service to be implemented (protocol document) (C1), they feel their scope/duties has been legitimised by the programme (C2) and are more likely to be comfortable to assert their role in implementing the pathways (O) and carry out professional negotiations to facilitate implementation (O).	*Yes, I suppose I would have to instil that (referral pathways) quite a bit when I started. There would have been a lot of inappropriate referrals...Maybe the previous podiatrist just took everything but then if you're expanding a service that's not possible or it's not going to be sustainable...So it would be very unfair for me to see all the low risks and the low risk can't attend in another hospital. (Pod#14, R4)*<br><br>*We needed something (programme) to say this is what has to be done, you know, that's what kind of made it easier going in implementing these changes, like "listen, it's not me, it's the programme, you have to do it". (Pod#12, R2)*
3	**Implementation; adjusting to fulfil immediate local need**	
3.1	**Implementation in primary care**<br>Depending on HCPs' resources (C) or experience in diabetes (C) they felt more or less supported by the programme (M) and therefore, adapted the programme to fulfil their immediate needs (O)	
3.1.2	*Selecting the 'tricky' ones*<br>Depending on practice's experience of diabetes (C) and resources (C) HCPs adjusted the programme as needed (M) either arranging for stable or complex patients to be seen by the DNS (O)	*I suppose the longer that I'm in practices they can see themselves the importance of just bringing the complicated to me and leaving the uncomplicated to themselves. (DNS#4, R4)*<br><br>*I suppose what it does is it takes <u>time</u> for somebody to sit down and go through their patients and pick out ones that need to be seen so really it*

		depends on who's doing it and how much time they have to go through those patients to see the ones that need to be seen. (DNS#13, R1)
3.1.3	*'Resource training' or 'doubling up'*<br>Practices which lacked resources (C) judged that the best way to use the integrated DNS service to meet their needs (M) was for the practice nurse not to take part in clinics with the integrated DNS (O)	*She [integrated DNS] is in there now and she knows it all so we don't need to..." and the practice nurse then will be pulled into doing all her other jobs while it's going on…..They don't want to make the time because... well, I'm there. I'm seeing the patients. So, they see that it's doubling up on two staff. (DNS#4, R4)*<br><br>*Our Practice Nurse tends to sit in with her, and, I mean, I think is gradually learning the ropes (GP#19, R2)*<br><br>*Some of the busier practices, no [the practice nurse does not sit in with me], not always, they will book the patients in but they cannot afford the practice nurse hours on a morning or an afternoon to be spent doing it. (DNS#6, R4)*
3.2	**Implementation in secondary care**<br>Depending on familiarity with the role of the integrated DNS and podiatrists among existing HCPs (C), resources (C) and existing professional boundaries in secondary care (C), new practitioners recruited by the programme and secondary care practitioners made judgements (M) about how to best adapted the role to fulfil immediate service	

	needs (O).	
3.2.1	*Professional boundaries*<br>Where there are established MDTs with good working relationships or receptive secondary care practitioners (C), podiatrists felt more supported and empowered (M) to implement the pathways (O).<br><br>Once the podiatrist was embedded in a MDT (C), it was more likely that they will work together around gaps in pathways (M) to implement modified pathways that are feasible (O)	*A lot of the girls that I know wouldn't have support from the diabetes consultant at podiatric level...Taken almost a year to get it up and running. If I wasn't attached to [X hospital], we wouldn't have this. It's because they're very supportive towards me. That's on a personal level as well. So I can directly access the podiatrist there or the professors in the team. (Pod#14, R3)*<br><br>*Whereas, thank god, all our consultants are interested in the foot, the diabetic foot...but you know the thing is we worked on that as well and there is a lot of give and take, you know, we will do things for them as well that we probably shouldn't be doing but you know it's communication I think (Pod#11, R2)*<br><br>*It works really well because we have all the facilities here, we have a consultant, we've got the diabetes nurses, we've got an orthotist, we've tissue viability, we have a dietician. We also even have a consultant, a vascular surgeon and he started maybe the year after, He's only here on a monthly basis, though...We've had to make our own pathways. There was no internal pathway obviously to do…so I developed a pathway when I*

		started as to how people would refer to the service and I suppose let them know the service was here as well. (Pod#2, R4)<br><br>*They have to private. And that's the thing, we can't be telling them who to go to because we don't have anyone specifically to send them to. So what we have been trying to do, but it's not really…we haven't really got much from it, is linking up with the likes of Navan and Cavan. You know, the actual hospitals outside of Dublin. Because we have a lot of people coming from outside of Dublin as well. (Pod#12, R2)*<br><br>*what we've got is [podiatrist] as a link for the whole lot. So, the community podiatrists send them in to her, and then, like today, she rang me twice this morning to say that she's got two people over here that look like they've osteomyelitis, so I came over. We do a foot round once a week with the antimicrobial pharmacist, diabetes nurse, me, podiatrist, and we see all the in house troublesome diabetic feet...We would do our own everything, except vascular...We've changed the pathway. We've made the pathway come here. We will refer then up to vascular if we need it, but they usually make a recommendation of come back here for the amputation, or not, or whatever. (Endo#1, R4)*

	Where strict professional boundaries existed (C), podiatrists may have felt it was futile (M) and they implemented the service elsewhere (worked around and sought out interested parties) (O).	*The doctors here don't do foot screening… And this comes from the consultant… It's not their job to do foot screening. (DNS#5)*<br>*If I didn't have X hospital and that direct access, this wouldn't work, because obviously our vascular would be covered by X and there is very little correspondence. I have broached the subject on setting up a similar MDT ... and there's no interest. We have very different views on that. (Pod#14, R3)*<br><br>*I'd say just getting ourselves out and educating, trying to show them that… Get you nearer to them, and show what we can do. I would say, if you don't agree with what they say... Well, personally, if I don't agree with it, I will question them. I won't say, 'You're the doctor, you're the consultant, okay.' I will ask again, 'Are you sure, for x, y and z?' And if they still say no, that's fair enough, you had the discussion, but I think it's...Doctors are still seen as demigods as such in Ireland…but if they're not interested in it anyway, then it's beating your head against a brick wall...There are obviously a few that don't have the interest in us as well, but because we have a team that are, we just stick to those. (Pod#11, R2)*
3.2.2	*Filling in service gaps*<br>In hospitals which lacked DNS resources (C) with limited control over the patient cohort (limited appointment slots) (C) secondary care practitioners judged the best way to use the DNS service ('opportunism') (M) was to have them fill in gaps in	*So now what we end up doing is actually putting her into our clinics, so to save one of our other nurses for the other patients seeing as we're short on nurses. (Endo#2, R1)*

	existing service (O) and serve another part of the team (same role as other hospital DNS) which does not fully align with the intended delivery of the new integrated DNS role (O)<br><br>Hospitals which had extra nurse resources (C) and some control over the patient cohort (dedicated appointment slots; type 2 clinics) (C) judged the best way to use the DNS service (M) was for them to see type 2 only or community patients which is more in line with the model as intended (O)	*We try to get her down in clinic and on the days that her patients are coming* ***[But that's not always possible?].*** *No, because it's a full clinic, so anything can randomly pitch in. (Endo#1, R4)*<br><br>*Well, we were kind of basically just told that it was one day per week, and that it was up to ourselves to decide what was the best way to run it. It seems she's working as part of the team, and that's... It's not any different to anybody else being on that on a particular day (DNS#7, R4)*<br><br>*There's four diabetes clinic and nurse specialists now, so that's quite an amount compared to years ago there was one. So that has exploded. (DNS#5, R3)*<br><br>*So sometimes she will bring in one or two patients in the afternoon and see them here. (DNS#5, R3)*
3.2.3	Given resource demands (C) and depending on the availability of existing community services (C), podiatrists made judgements about how to provide the best service (M) in order to meet the immediate service needs (O).	*To get this programme up and running you really need a lot more people on the ground. We're struggling at the ulcerated foot, the 'active foot' as it's called in the model of care…we're not even close to meeting what would be called for at the moderate or high-risk end. (Working group #4)*<br><br>*It's...being implemented but it's only being implemented in the short term. Its successes are noted but it's also reached its capacity in that there needs*

		to be more investment by the HSE into podiatrists in order to extend the scope of it...they need to be extended because the key personnel that are there are and now the backlog is starting for them. (Working group #2)<br><br>*I think it's [model of care] just not...hasn't been supported with staff...we're not adequately resourced. We try to provide the best service that we can with the resources available. (Endo#1, R1)*<br><br>*When you're seeing the high risks, you're firefighting. When you're seeing the actives, you're beyond that. So, you're just trying, preventing as little destruction as possible. (Pod#11, R2)*<br><br>*They have to go to private. And that's the thing, we can't be telling them who to go to because we don't have anyone specifically to send them to...We're continuing to see the high risk patients that could really be managed in community but they're swamping up our clinics in here as well. (Pod#12, R2)*<br><br>*We don't get referrals for high risk, they need to go to the community. At the moment, there aren't that many community podiatrists out there. Our main issue would be where do they go when they are healed, long term, to keep an eye on them more regularly? We do keep an eye on them, even*

		when they're healed…but we have to spread it out much further, because we have to prioritise our active ones here. (Pod#2, R2)<br><br>*On the whole, it is working well, but I think for the moderate risk patients, at the moment, we just can't see them as frequently as we should be. But definitely the moderate risks have been neglected, for the last year, just because our caseload of high risk patients has just gone through the roof. (Pod#20, R1)*<br><br>*Obviously since 2011, since the model of care, it has completely changed. It has a huge effect on our services. But the effect it had on us is that it's becoming a more of, what I would call, a high risk service. (Pod#2, R4)*<br><br>*The problem is some of the ones with moderate, you know if you send them, they're probably never going to get seen. Some areas don't seem to have enough podiatry support. So it's the ones in the middle, like if they're acute and complicated and they have an active foot problem, then they'll get seen in a hospital service but if they're somebody who's maybe poor with the old hygiene and nobody to cut the nails then unless they can pay for a service, they are probably not going to get a service in a lot of areas. (DNS#13, R1)*

*participants are numbered in order of when they were interviewed.
