# Supplementary material for: Adoption and Initial Implementation of a National Integrated Care Programme for Diabetes: A Realist Evaluation
Source: Int J Integr Care. 2022 Jul 14;22(3):3. doi: 10.5334/ijic.5815 (PMC9284993; doi:10.5334/ijic.5815)
Supplement: Additional Files. — Additional Files 1 to 6. [file ijic-22-3-5815-s1.zip › s1-ijic-5815_riordan/file6-ijic-5815_riordan.docx]

| **Complicated** | **425.1** | **207.3** | **228.0** | **440.0** | **320.7** | **152.7** | **181.1** | **523.2** | **154.1** |
| --- | --- | --- | --- | --- | --- | --- | --- | --- | --- |
| **Uncomplicated** | **171.9** | **108.3** | **160.0** | **166.2** | **196.4** | **115.3** | **71.4** | **51.7** | **153.1** |
| **Type 1** | **18.1** | **34.3** | **25.0** | **21.8** | **8.4** | **4.4** | **6.9** | **27.9** | **7.4** |

**Figure 1:** Complicated type 2 diabetes, Uncomplicated type 2 diabetes and type 1 diabetes patient episodes per WTE by Community Healthcare Organisation (annual)*

*Data were collected in between January and December 2017 by 29^[[1]](#footnote-1)^ of 30 (28.46 WTE) DNS posts. These 29 posts represent 27.46 WTE.

**References**

44. Riordan F, McHugh SM, Murphy K, Barrett J, Kearney PM. The role of nurse specialists in the delivery of integrated diabetes care: a cross-sectional survey of diabetes nurse specialist services. BMJ open. 2017;7(8).

51. McHugh S, Tracey ML, Riordan F, O’Neill K, Mays N, Kearney PM. Evaluating the implementation of a national clinical programme for diabetes to standardise and improve services: a realist evaluation protocol. Implementation Science. 2016;11(1):107.

54. Riordan F, McGrath N, McHugh S.M., Kearney P.M., Twamley H, N S. Overview of Activity Data in Primary Care from Clinical Nurse Specialist (CNSp) Diabetes Integrated Care Group National Clinical Programme for Diabetes (NCPD); 2018.

60. Riordan F, McHugh SM, N. M, Kearney PM. ‘Sink or Swim’. Adapting to support the delivery of integrated diabetes care: experiences of clinical nurse specialists. International Journal of Integrated Care, 19 (2).

1. 31 DNS fill these posts; there are two job-sharing posts in CHO 9; 32 CNSp were in post but 31 were available to return data [↑](#footnote-ref-1)
