# Supplementary material for: Adoption and Initial Implementation of a National Integrated Care Programme for Diabetes: A Realist Evaluation
Source: Int J Integr Care. 2022 Jul 14;22(3):3. doi: 10.5334/ijic.5815 (PMC9284993; doi:10.5334/ijic.5815)
Supplement: Additional Files. — Additional Files 1 to 6. [file ijic-22-3-5815-s1.zip › s1-ijic-5815_riordan/Ref-ijic-5815_riordan.docx]

44. Riordan F, McHugh SM, Murphy K, Barrett J, Kearney PM. The role of nurse specialists in the delivery of integrated diabetes care: a cross-sectional survey of diabetes nurse specialist services. BMJ open. 2017;7(8).

51. McHugh S, Tracey ML, Riordan F, O’Neill K, Mays N, Kearney PM. Evaluating the implementation of a national clinical programme for diabetes to standardise and improve services: a realist evaluation protocol. Implementation Science. 2016;11(1):107.

54. Riordan F, McGrath N, McHugh S.M., Kearney P.M., Twamley H, N S. Overview of Activity Data in Primary Care from Clinical Nurse Specialist (CNSp) Diabetes Integrated Care Group National Clinical Programme for Diabetes (NCPD); 2018.

60. Riordan F, McHugh SM, N. M, Kearney PM. ‘Sink or Swim’. Adapting to support the delivery of integrated diabetes care: experiences of clinical nurse specialists. International Journal of Integrated Care, 19 (2).
